# The confidence and competence of primary school staff to administer an adrenaline auto-injector

**DOI:** 10.1007/s00431-024-05562-y

**Published:** 2024-04-12

**Authors:** P. Donovan, P. O’Connor

**Affiliations:** School of Medicine, University of Galway, Galway, Ireland

**Keywords:** Anaphylaxis, Allergy, Adrenaline, AAI, School staff, Ability

## Abstract

School teachers are often inadequately prepared to use an adrenaline auto-injector (AAI), resulting in potentially dangerous treatment delays. The purpose of this study was to assess the observed competence, and self-reported confidence, of primary school teachers in the Republic of Ireland (RoI) to use an AAI. An evaluation of whether there was a link between confidence and competence was also assessed. Teachers from four primary schools in the RoI completed a questionnaire to assess their prior level of experience, training, and confidence levels with AAI administration. The four steps in administrating trainer AAI to a mannequin simulator were then assessed. A total of 61 teachers participated (out of a population of 80). The mean self-reported confidence was 1.82 out of 5 (SD = 0.96). There was no significant difference in confidence between trained and untrained participants (*U* = 240.5, NS). Participants who had received AAI administration training performed significantly more of the steps correctly (mean = 3.85, SD = 0.95) as compared to those who had received no training (mean = 2.97, SD = 1.10; *U* = 180.5, *p* = 0.008). There was no correlation between confidence in administrating AAI and the percentage of steps in the procedure performed correctly (rho =  −0.17, NS).

*  Conclusion*: Improvements in readiness to administer AAIs can be achieved through the application of more effective approaches to teaching clinical skills, changes to school policies and practices, and consideration of the design of AAIs in order to make their operation safer and simpler. It is important that teachers have the confidence and competence to safely administer an AAI.

**Table:** 

**What is Known:** *• Poor ability in adrenaline auto-injector use seen across population groups—healthcare professionals, patients, carers, and school staff* *• Training in the use of adrenaline auto-injectors has positive impact on competency*	
**What is New:** *• Irish school teachers show poor levels of competency in adrenaline auto-injector use* *• No observed correlation between reported confidence and competency*

**What is Known:**

*• Poor ability in adrenaline auto-injector use seen across population groups—healthcare professionals, patients, carers, and school staff*

*• Training in the use of adrenaline auto-injectors has positive impact on competency*

**What is New:**

*• Irish school teachers show poor levels of competency in adrenaline auto-injector use*

*• No observed correlation between reported confidence and competency*

## Introduction

Anaphylaxis is a severe life-threatening hypersensitivity reaction, with acute onset and multisystem involvement. A leading cause of anaphylaxis is immunoglobulin E (IgE)-mediated food allergy [1]. The estimated prevalence of food allergies is between 1.4% and 3.8% in school children [2]. The signs and symptoms of anaphylaxis are varied, with potential to involve the skin, respiratory and gastrointestinal tracts, and the cardiovascular and neurological systems [3]. The recommended treatment for anaphylaxis is adrenaline, given via the intramuscular route, usually delivered in the community with an adrenaline auto-injector (AAI) [4]. Early administration of adrenaline is recommended once anaphylaxis is recognised. However, there are often delays in treatment of anaphylaxis especially in the community setting [5]. Delay in administration can result in an increased risk of death [6, 7].

Due to the large amount of time children spend in education, it is not uncommon for anaphylaxis to occur within a school setting. Reported incidence of anaphylaxis in the school setting varies from 10% to 16% [5, 8, 9], with almost half of these occurring in children without a prior known food allergy [10]. However, research on the competence of school staff to use AAI is limited. A study carried out in Germany in the preschool setting found that levels of competence in AAI use were low—although there was a positive improvement following an educational intervention [11]. A weakness of some of the research in the area is that data on the confidence to use an AAI has tended to be based upon self-report [12, 13], rather than through observation of actual performance. This is a large limitation as it has been shown that people with a low ability in a specific area can give overly positive assessments of their ability. This phenomenon has been described as the Dunning-Kruger effect [14]. This effect has been identified in a range of studies—including with medical profession [14, 15].

Our study will address the shortcomings of previous research by: (1) assessing the observed and self-reported confidence of school staff in the Republic of Ireland (RoI) to use an AAI; (2) evaluating if there is an association between confidence and competence; and (3), based on the findings, make recommendations for future research and practice.

## Methods

### Design

This study used survey and observation methods. Data was collected from March to April 2023.

### Ethical approval

Ethical approval was received from the Research Ethics Committee of College of Medicine, Nursing and Health Sciences, University of Galway, on 3 March 2023 (ref: 22.23–066). All participants provided signed informed consent. The study was performed in accordance with the ethical standards as laid down in the 1965 Declaration of Helsinki.

### Participants

Participants were teachers or special needs assistants from four primary schools in Cork City in the RoI.

### Recruitment

Participants were recruited by first contacting primary school principals via email and inviting teaching staff in their school to take part in the study. Written information was provided about the study and the information was disseminated to teaching and special needs assistants in the school. Six school principals were contacted and four agreed that staff in the school could be recruited. Staff who wished to participate in the study responded via a survey link which included the participation information leaflet, the inclusion and exclusion criteria, and an electronic consent form.

### Procedure

The data collection was carried out by a medical doctor (PD). PD is a paediatric trainee and experienced in prescribing AAIs.

On the day of data collection, the participants were made aware that they would be scored on their ability to administer the AAI correctly. A room was provided in the recruited schools to complete the data collection. The participants completed a background questionnaire to provide information on their years as a teacher, past training they received on the recognition of anaphylaxis and/or the administering an AAI (how long, and when), and their confidence in correctly administering an AAI from 0 (not confident at all) to 5 (completely confident).

Once the participants had completed the questionnaire, they were observed administering a Jext^®^ AAI trainer on a clothed low fidelity Laerdal^®^ paediatric mannequin. The instructions given to the participant were “to take the Jext^®^ trainer pen and administer it as you see fit, and inform the assessor as to when you feel all steps have been completed”. The Jext^®^ AAI trainer pen was selected following discussions with allergy specialists about what brand of AAI should be used in the study. The rationale for choosing the Jext^®^ AAI was that the shelf life of this AAI is longer than other AAIs available in the RoI. This is an important consideration as the majority of people in the RoI must pay for a prescription. As a result, we were told that the Jext^®^ AAI is being prescribed with greater frequency than other AAIs in the RoI.

There is no agreed scoring system for correct AAI administration. A 2-step guide to the procedure is displayed on the body of the Jext^®^ trainer and real Jext^®^ AAIs. To allow for ease of assessment, these steps were broken into four sub-steps similar to a scoring system used by a previous study that assessed the use of AAI with school staff [16]. The steps were:

1. Cap removed.
2. Device placed against the outer part of the thigh at the correct angle.
3. Click heard.
4. Device held for 10 seconds against the thigh.

The observer recorded whether each step was performed correctly/incorrectly, and the total time to complete the procedure. Participants who had completed the assessment were asked to refrain from conversing with other participants so as not to affect the results of the study. Following the completion of the observation, the observer (PD) provided feedback on the performance of each participant and answered any questions. Following all data collection, PD provided a 20-minutes-long face-to-face training session to staff in the school on anaphylaxis.

The procedure and questionnaire were piloted with 7 medical students. Changes made to the procedure included the observer being a short distance away from the participant to ensure that the assessment recordings could not be seen by the participants, and some minor changes were made to the wording of the questions about prior training.

### Sample size

The population of teachers and special needs assistants in the four primary schools recruited into the study was 80 people. To achieve a margin of error of 8% and confidence level of 95%, the recruitment required 52 participants.

### Statistical analysis

The analyses were carried out using IBM SPSS Statistics (Version 27). A Mann–Whitney *U* test was used to examine if there were differences between participants who had received prior training and those who have not in their confidence and ability to use the AAI. Spearman’s rho was used to assess for possible correlation between percentage of steps performed correctly and prior training.

## Results

### Participants

There were a total of 61 participants from 4 state run primary schools. This results in a margin of error of 6.2%. The participants in the study compromised of 82% (50/61) teachers and 18% (11/61) special needs assistants. A total of 85% (52/61 of the participants) were women. Participants had a median of 12.9 years (range 2–38 years) working in their current role. All participants completed the study.

### AAI and anaphylaxis recognition training

A total of 80% (49/61) of participants had never received any training in recognition of anaphylaxis, and 77% (47/61) reported receiving no training in AAI use as a teacher. Only one of the four participating schools had arranged for training for their staff, all other training had been either given by a caregiver or at a prior school. For those who had received training, the median time since training was 3 years (range 0.5–15 years) in the recognition of anaphylaxis and 2.5 years (range 0.5–13 years) in AAI use. The median training time reported was 30 min (range 10–60 min). Only 3% (2/60) participants had ever administered an AAI in real life, with 32% (19/60) reporting administrating an AAI in a training session.

### Self-reported confidence

The self-reported confidence in correctly administering an AAI was low for the majority of the participants (see Table 1). The mean self-reported confidence was 1.82 out of 5 (SD 0.96). There was not a significant difference in the confidence in using an AAI between trained and untrained participants (*U* = 240.5, NS; see Table 1).

**Table 1:** Self-reported confidence in correctly administrating an AAI

**Self-reported confidence**	
	Percentage (*n*)
Not confident	50.8 (31)
Slightly confident	21.3 (13)
Somewhat confident	23.0 (14)
Fairly confident	4.9 (3)
Complete confidence	0 (0)
	Mean confidence (SD)
Trained	2.14 (1.02)
Untrained	1.72 (0.93)
All	1.82 (0.96)

### Observed AAI performance

Table 2 outlines the mean percentage of participants who performed each of the five steps correctly. The most common error was not holding the device in place for 10 seconds. The mean time to complete the procedure was 24.21 seconds (SD = 12.95). For the six participants who performed the procedure correctly, the mean time to complete was 31.1 seconds (SD = 11.86). The number of steps performed correctly between those participants who had received AAI training was significantly higher than those who had not (see Table 2).

**Table 2:** Comparison between those trained and untrained in AAI use

**Step performed**	**Trained** % (*n*)	**Untrained** % (*n*)	**All** % (*n*)	**Specific issues** (*n*)
Number of participants	14	47	61	-
Removal of safety cap	85.7 (12)	61.7 (29)	67 (41)	• Safety cap removed partially correctly (20)
Placement of device to outer thigh at correct angle	78.6 (11)	80.9 (38)	80 (49)	• Elsewhere on thigh (6) • Elsewhere on body (5) • Not on body (1)
Black tip placed toward mannequin	100 (14)	83.0 (39)	87 (53)	• Device placed upside down (8)
Click heard	85.7 (12)	53.2 (25)	61 (37)	• Click not heard (24)
Device held in place for 10 seconds	35.7 (5)	19.1 (9)	23 (24)	• 3 s (20) • > 3 s and < 10 s (27)
All steps performed correctly	21.4 (3)	6.4 (3)	9 (6)	-
Mean number of steps performed correctly (SD)	3.85 (0.95)	2.97 (1.07)	3.18 (1.10)	• *U* = 180.5, *p* = 0.008 • Effect size (*d*) = 0.87

### Confidence versus performance

There was no correlation between confidence in administrating AAI and the percentage of steps in the procedure performed correctly (rho =  − 0.17, NS). It is interesting to note that for the six participants that performed all of the steps correctly, their mean confidence was only 1.67 out of 5 (SD = 1.21).

## Discussion

Food allergies are increasingly common in children [2]. Therefore, teachers are more likely than ever to be faced with the challenge of managing a pupil with anaphylaxis. However, teachers are often inadequately prepared to use an AAI, resulting in potentially dangerous treatment delays [4, 7, 17, 18]. Our study evaluated the confidence of teachers to use an AAI, the competence of teachers in performing the steps required to administer an AAI, and assessed if there is a link between confidence and competence.

The majority of the participants in our study reported little or no confidence in their ability to correctly use an AAI. This was even the case for the six participants who correctly performed the procedure, and for those respondents who had attended AAI training. Our findings agree with other studies that have found that even with training, parents and caregivers have low levels of confidence and high levels of anxiety in correctly using an AAI [19–21]. This lack of confidence may lead to AAI use hesitancy in which treatment is delayed because caregivers are afraid to use the device. Evidence for this suggestion comes from a survey in which, of those respondents (parents or people with food allergies) who had experienced anaphylaxis, only a third had administered an AAI. The majority of the respondents reported that treatment was not given until the person experiencing anaphylaxis got to the Emergency Department [22]. This delay is concerning as early administration of adrenaline is important in the treatment of anaphylaxis, and a delay in administration has been found to be associated with an increased risk of death [23]. Therefore, it is important to address the lack of confidence that may lead to hesitancy in administering an AAI.

Only six participants in our study performed every step correctly in the administration of the AAI. The most frequently observed error was not holding the device in place for 10 seconds. Almost a third of participants held the device in place for less than 3 seconds. Eight participants incorrectly inverted the AAI such that they would have experienced a digital self-injection if a real AAI was used. Our findings are similar to other studies that have observed AAI administration with a teacher population [11, 16]. Although there was not an impact of prior training on confidence, there was a large effect of training on the number of steps performed correctly. The positive effect of training on AAI use is consistent with findings from other studies that have examined the impact of AAI training for preschool staff [11], medical staff [24], caregivers [25], and patients [26]. To some extent, the impact of training was a little unexpected. None of the participants had received AAI administration training in the last 6 months, and research has demonstrated skill decay in AAI administration 4 to 12 weeks after training [11]. Another interesting finding was that three of the participants who completed all of the steps correctly had not received any AAI training and were able to deliver the AAI successfully by using the instructions printed on the body of the device. Therefore, at least in simulated setting, there is utility in these instructions if they are noticed and used.

Our study did not find a significant correlation between observed performance and self-reported confidence. Therefore, the Dunning-Kruger effect [14]—in which people tend to overestimate their performance—was not observed. Our findings contrast with an evaluation of AAI training carried out with preschool teachers that found that the effect an education intervention had on confidence remained high even 4 to 12 weeks after the AAI training. However, the percentage of correct AAI administrations was 3% before the training, 35% immediately after the training, and 16% 4 to 12 weeks after the training [11]. Either way, both studies demonstrate that, although determining competency based upon self-reported confidence is attractive as it requires little resources to complete, it is also generally invalid. Training in AAI administration would appear to be necessary, but not sufficient, for teachers to be confident in administering an AAI.

It is possible to make recommendations based upon the findings of our study for researchers, schools, education authorities, and AAI manufacturers. It is suggested that researchers consider the efficacy and efficiency of different approaches to training on both observed competence and confidence. Precision teaching using simulation is suggested as an educational approach to build fluency in AAI administration. Once fluency has been attained, it is possible to complete the task with little conscious effort—even when the training was completed a long time ago [27]. Precision teaching, utilising simulation, has been found to be an efficient and effective approach to building fluency in a number of clinical skills (e.g. venepuncture [28], lumbar puncture [28]). Schools and education authorities must recognise the increased likelihood of teachers encountering anaphylaxis and develop robust policies, and training, so that teachers are adequately prepared to manage anaphylaxis. Consideration should also be given to keeping AAI devices in schools. Although this is not done in the RoI, this is the case in countries such as USA, Canada, and England [29]. Teachers would then only have to be trained to use the AAI that is stocked by the school. This would address the issue of not knowing how to use all four types of AAI prescribed in Ireland. It would also mean that the teachers could administer a second dose if required. A survey of adults found that 82% did not carry a second AAI—despite the fact that a second dose of adrenaline is commonly required [5]. Finally, it is suggested that AAI manufactures should use the principles of human factors engineering to consider how the design of AAI could be changed in order to make the devices easier (and safer) to administer and address issues with variability in operations between devices. To illustrate, the voice-prompted AUVI-Q device, developed with patient input, has shown promising results in comparison to other devices—even with device-naive users [30, 31].

## Limitations

There are a number of limitations that should be acknowledged. Only staff from four primary schools participated in the study from one city in the RoI. Therefore, the generalisability of the findings, particularly outside the RoI, could be questioned. The administration of only one AAI was assessed in this study. However, similar levels of competency have been found in using both the Jext^®^ and Epipen^®^ AAIs [30], so we believe that the findings generalise to other AAIs. We only assessed the ability of the participants to use an AAI. We did not assess their knowledge of the symptoms, or management, of anaphylaxis. Finally, our recruitment method could have led to selection bias. Previous research has shown that if teachers are aware that they are going to be assessed administrating an AAI, then their performance is superior to teachers who were not informed of the assessment [16]. However. the ethics board did not deem it appropriate to blind the participants to the assessment. Therefore, the level of performance may be lower in the general teacher population.

## Conclusion

Due to the increasing prevalence of food allergies. teachers are more likely than ever to be faced with the challenge of managing a pupil with anaphylaxis. However, although not a complex task, teachers largely lack the confidence and competence for AAI administration. Therefore, action is required to ensure teachers are confident and competent to manage this potentially life-threatening condition. This can be achieved through the application of approaches: teach clinical skills such as precision teaching, changes to school policies and practices, and consideration of the design of AAIs in order to make their operation simpler.

## Data Availability

Available upon request to the corresponding author.

## Abbreviations

AAI: Adrenaline auto-injector
RoI: Republic of Ireland
IgE: Immunoglobulin E
*n*: Number
SD: Standard deviation
